# Deep learning applied to hyperspectral endoscopy for online spectral classification

**DOI:** 10.1038/s41598-020-60574-6

**Published:** 2020-03-03

**Authors:** Alexandru Grigoroiu, Jonghee Yoon, Sarah E. Bohndiek

**Affiliations:** 1 Department of Physics, University of Cambridge, JJ Thomson Avenue, Cambridge, CB3 0HE United Kingdom; 2 CRUK Cambridge Institute, University of Cambridge, Robinson Way, Cambridge, CB2 0RE United Kingdom

**Keywords:** Scientific data, Applied optics

## Abstract

Hyperspectral imaging (HSI) is being explored in endoscopy as a tool to extract biochemical information that may improve contrast for early cancer detection in the gastrointestinal tract. Motion artefacts during medical endoscopy have traditionally limited HSI application, however, recent developments in the field have led to real-time HSI deployments. Unfortunately, traditional HSI analysis methods remain unable to rapidly process the volume of hyperspectral data in order to provide real-time feedback to the operator. Here, a convolutional neural network (CNN) is proposed to enable online classification of data obtained during HSI endoscopy. A five-layered CNN was trained and fine-tuned on a dataset of 300 hyperspectral endoscopy images acquired from a planar Macbeth ColorChecker chart and was able to distinguish between its 18 constituent colors with an average accuracy of 94.3% achieved at 8.8 fps. Performance was then tested on a set of images simulating an endoscopy environment, consisting of color charts warped inside a rigid tube mimicking a lumen. The algorithm proved robust to such variations, with classification accuracies over 90% being obtained despite the variations, with an average drop in accuracy of 2.4% being registered at the points of longest working distance and most inclination. For further validation of the color-based classification system, ex vivo videos of a methylene blue dyed pig esophagus and images of different disease stages in the human esophagus were analyzed, showing spatially distinct color classifications. These results suggest that the CNN has potential to provide color-based classification during real-time HSI in endoscopy.

## Introduction

Hyperspectral imaging (HSI) refers to the collection of both spatial (*x*, *y*) and spectral (*λ*) information from a sample. The 3-D data structure, known as a hypercube, is a stack of 2-D images, each capturing information from a narrow spectral range^1–3^. The application of HSI for *in vivo* disease diagnosis within the human body, for example in the gastrointestinal tract, requires the HSI system to be paired with a flexible optical fibre endoscope to access internal body cavities. Challenges arise from this pairing due to: motion of the lumen and flexible endoscope during hypercube data acquisition; and image artifacts resulting from variable working distances as well as cladding structures present when using a multi-core optical fiber bundle endoscope^4,5^. To overcome these hardware challenges, recent efforts have focused on achieving a compromise between spatial, spectral and temporal resolutions^6,7^. In particular, a spatial-scanning hyperspectral endoscope (HySE) has recently been reported that obtains wide-field color (RGB) images, and line-scan hyperspectral data from the mid-line of the field-of-view, simultaneously at over 20 fps as the endoscope sweeps across the lumen^4^. The color images are used to correct for image artifacts and compile a 3-D hypercube with high spatial and spectral resolution from the line-scan data.

Real-time HSI systems, such as in HySE, inherently generate a large volume of multidimensional data. To achieve meaningful clinical deployment, these data must be rapidly analyzed in order to provide real-time feedback to the operating endoscopist for clinical decision making. Many techniques have been developed and used in biomedical optics for the analysis of hyperspectral images, including conventional multivariate statistical methods as well as more recent developments in advanced learning algorithms^8^. Based on these analyses, HSI has shown promise in a range of biomedical applications by capturing subtle changes in the physiology, morphology and biochemistry of tissues under pathologic conditions^1,2^. For example, Pearson correlation analyses compare a spectral image to a library of known spectra; the highest correlation coefficient gives the segmentation result, which has previously been applied for discrimating colonic adenomas from normal mucosa^9^. A similar library-based method is spectral unmixing, which decomposes a mixed pixel into a collection of known spectra and has shown good performance in the characterization of burn lesions^10^ or the investigation of age-related macular degeneration^11^. Unfortunately, Pearson correlation analyses and spectral unmixing are relatively slow, which presents difficulties for online classification. Support vector machines (SVMs) maximize the distance between a decision boundary and members of different classes and have been applied successfully in the classification of brain tumors^12^. However, due to SVMs being binary classifiers, their speed is poor when used in the online segmentation of multi-class problems. Finally, advanced learning algorithms have been shown recently to combine speed with accuracy^13–17^, which makes them promising candidates for online evaluation of HSI data. Such algorithms have seen a wide range of applications in the field of hyperspectral imaging of tissue, from the prediction of spectral signals from white light images^14^ to the extraction of specific measures of cancer progression^14,16^. For direct image interpretation, techniques such as generative adversarial networks^15^ and fully-convolutional neural networks^15,17^ have achieved success, with pixel-wise classifiers also showing high performance^13^. Implementations of the latter two architectures have been of particular interest in the development of this work.

Here, we tested the performance of a deep-learning algorithm based on a color-classification approach for the real-time analysis of endoscopic HSI data acquired using the aforementioned spatial-scanning HySE system. By directly analyzing the line-scan data, we overcome challenges in motion artifacts, which could enable real-time visualization of information derived from the HSI data. Color-based classification was chosen considering the current use of subtle color changes for interpretation of standard white-light endoscopy. We trained a five-layered convolutional neural network (CNN) using a standard Macbeth ColorChecker color chart and compared the performance of the CNN with conventional spectral analysis methods using a subset of 4 of the 18 available colors on the chart. We then demonstrated the feasibility of the CNN for rapid classification of the full color chart under endoscopic imaging conditions, also deforming the color chart into the shape of a lumen. Finally, we applied the CNN to *ex vivo* data obtained from an intact pig esophagus and human biopsies taken from the esophagus. The CNN proved to be the best candidate for classifying HySE data and suggests a deep-learning approach may facilitate the online analysis of biological tissues during endoscopy.

## Results

### Color-based classification and test data

**Figure 1:**
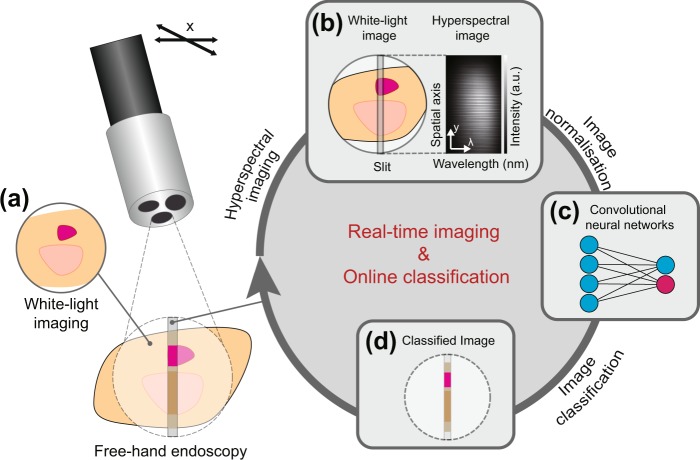
Schematic of the proposed method for classifying HySE data. (**a**) A white-light image is captured in parallel with the hyperspectral data during the endoscopic procedure. (**b**) Hyperspectral data is collected from a slit centered in the capture area, with the resulting data having spatial and spectral dimensions. (**c**) The captured hyperspectral data is preprocessed and fed into the CNN. (**d**) The learning algorithm classifies the hyperspectral data and the result is overlaid over the white-light image. The classification process is continuous with the endoscope being subjected to free-hand movement.

An overview of the method developed for color-based classification of line-scanning hyperspectral endoscopy (HySE) data is depicted in Fig. 1. Both white light images and hyperspectral line-scan data are acquired as the endoscope is moved laterally (Fig. 1a,b). The captured hyperspectral data is then analysed using the convolutional neural network (CNN, Fig. 1c) or alternative classification method, before the classification result is displayed as an overlay on the white-light image (Fig. 1d).

Five different datasets are used in the present study (see Table 1 and Methods). The first three datasets consist of hypercubes taken from the Macbeth color chart, while the latter two come from measurements of tissue samples. In each case, the exact dimensions of the recorded data varies depending on: the area scanned (*x*); the grating used (*λ*); and the number of replicate samples included (*n*). The five datasets are described below, with details on their acquisition conditions, data dimensions and the resulting classification speed of the CNN given in Table 1.

**Table 1:** Dataset parameters and CNN classification speeds. Column definitions: Acquisition, motorized stage or freehand motion used for spatial scanning; Grating, lines/*mm*; n, number of technical replicates performed, except for tissue biopsies where it is the number of biopsy samples imaged; x, number of spatial steps resulting in 2D spatial-spectral images employed for training; y, spatial size of the measured line on the spectrometer camera in pixels (each of dimension 16 *μm* × 16 *μm*); *λ*, spectral dispersion on the camera in pixels; CNN speed, potential classification speed calculated from the time taken for analysis when the CNN is applied to the dataset.

Dataset	Acquisition	Grating	*n* (samples)	**x** (spatial steps)	**y** (pixels)	**λ** (pixels)	CNN speed (fps)
Four color	Motorized	300	4	105	512	1209	8.8
Planar	Motorized	300	3	247	421	1210	7.0
	Motorized	50	3	160	421	198	7.1
Tube	Motorized	300	2	140	421	1210	7.0
	Motorized	50	2	140	421	198	7.0
Pig esophagus	Freehand	50	1	97	512	198	7.1
Tissue biopsies	Motorized	300	12	6512	378 - 560	121	6.9

- The *four color dataset* comprises 2D spatial-spectral images obtained from a single 4 color section of the Macbeth color chart, with data acquired under different illumination conditions and imaging angles, varying between 70° and 90°.
- The *planar dataset* comprises 2D spatial-spectral images of different fields of view and locations across the Macbeth color chart, encompassing all 18 colors of the color chart.
- The *tube dataset* comprises 2D spatial-spectral images of a Macbeth color chart bent and placed inside a Berzelius beaker for support.
- The *pig esophagus dataset* comprises 2D spatial-spectral images of a pig esophagus obtained from Medical Meat Supplies (UK), where the blood had been drained and the lumen had been dyed with methylene blue. Each image represents the hyperspectral measurement from a frame in the captured video of the pig esophagus^4^.
- The *tissue biopsies dataset* comprises 2D spatial-spectral images of human tissue biopsies (n = 3 patients; n = 12 biopsies) from the gastrointestinal tract obtained during a previous study^4^.

### Four color dataset

To compare the performance of the different classification methods, a restricted four color dataset obtained from a single section of the Macbeth color chart was first employed, with the different learning techniques being trained on the subset of training colors corresponding to the four color dataset. Results are summarised in Table 2. The Pearson correlation analysis shows the poorest accuracy, while the spectral unmixing methods show the longest analysis time and hence the slowest frame rate. SVM shows improved performance over these methods, however, an increase in the number of binary classifications presents scaling problems in terms of the analysis time. The CNNs outperform all other algorithms tested in terms of both the classification accuracy and the achievable classification frame rate (~8.8 fps) for real-time application. Variation in the classification time between the two CNN techniques is negligible, although the pixel-wise architecture outperforms the alternative slice-wise CNN implementation by a significant margin in terms of average classification accuracy. Given the poor scalability of the conventional spectral analysis methods to data with a higher number of classes, and the higher demand for training data for the slice-wise CNN when scaling the number of colors, the pixel-wise CNN was taken forward and tested in more complex scenarios for the remainder of the paper.

**Table 2:** Quantitative performance comparison of the spectral classification algorithms. Column definitions: Accuracy, mean accuracy calculated over the four color dataset; Speed: potential classification speed calculated from the time taken for analysis when the specific method is applied to the test dataset; Training time, the time it takes to train the algorithm on the four colour dataset.

Algorithm	Accuracy (%)	Speed (fps)	Training time (min)
Pearson correlation analysis	85.3	1.1	11
Supervised spectral unmixing	90.6	0.4	51
Unsupervised spectral unmixing	90.4	0.3	51
Support vector machines	91.2	4.1	70
Pixel-wise CNN	94.5	8.8	360
Slice-wise CNN	90.7	8.9	275

### Planar dataset

**Figure 2:**
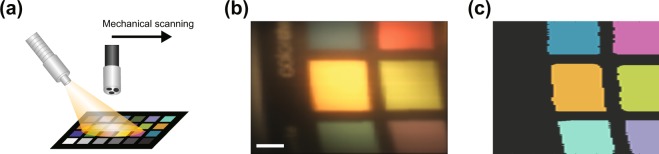
Classification results for the planar dataset, corresponding to a scan of a color chart area. (**a**) Schematic of the process of acquiring planar images. (**b**) Stitched version of the reference white-light image, corrected for the fibre artifacts and stitching artifacts. (**c**) Prediction map of the scanned area from the pixel-wise CNN. Note that the pink and purple classification labels are generated from the RGB color code provided by the color chart manufacturer, which are maintained under normal white light conditions but exhibit some color deviation during endoscopy, as seen in (**b**). Scale bar = 5 *mm*.

To quantify the performance of the pixel-wise CNN when classifying all colors in the color chart, larger regions of the Macbeth color chart (Fig. 2a) with different fields of view and orientations were analyzed. The reconstructed reference image from the wide-field color camera (Fig. 2b) involved median filtering of the individual line-scan segments to remove fiber bundle image artifacts and Gaussian blurring of the resulting image to remove stitching artifacts. The prediction result (Fig. 2c) shows excellent overlap with the wide-field reference image. The pink and purple colors in the stitched image appear different from the RGB values quoted by the manufacturer, arising due to the illumination on the sample leading to a change in hue. However, this does not affect the spectral analysis of the system. An average classification accuracy of 94.3% was obtained across all 407 different 2D spatial-spectral images at a speed of ~7 fps, with incorrect classification mostly occurring at the sharp edges of the color squares.

### Tube dataset

**Figure 3:**
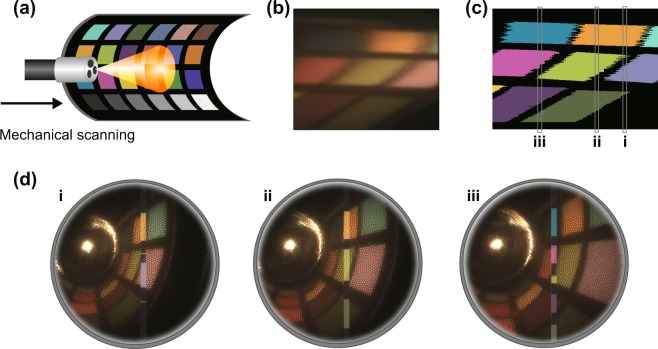
Classification results for the tube dataset, corresponding to a scan of a color chart area. (**a**) Schematic of the process of acquiring tube images. (**b**) Stitched version of the reference image, corrected for the fibre artifacts and stitching artifacts. (**c**) Prediction map of the scanned area. (**d**) Proposed real-time feedback for the slices (i, ii, iii) highlighted on the prediction map. A typical output is comprised of the white-light background image, with the classified hyperspectral data being shown in the line from which it was collected.

As the planar dataset is obtained with the endoscope positioned at a fixed angle (90°) and working distance (3 *cm*), it does not accurately model the imaging conditions encountered during endoscopic surveillance of the gastrointestinal tract. To test the performance of the CNN in a more realistic scenario with variable working distance and illumination angle, the color chart was bent into a cylindrical lumen (Fig. 3). The reconstructed reference image from the wide-field color camera (Fig. 3b) illustrates the distortions arising from the bending, yet the CNN classification results (Fig. 3c) still provide an average classification accuracy of 91.9% at ~7 fps. The 2.4% drop in accuracy arises primarily from the regions with higher working distance from the tip of the endoscope, due to colors blending together close to the edges of the color squares. The tube dataset also provides an opportunity to examine how the classified data might be presented to an endoscopy operator during real-time operation (Fig. 3d). In this case, rather than a fully reconstructed 3D hypercube being classified, each 2D spatial-spectral line is classified and then overlaid on the wide-field image.

### Pig esophagus dataset

**Figure 4:**
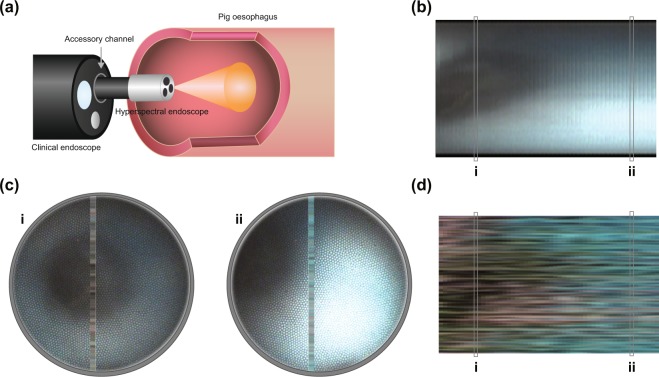
Classification results for the pig esophagus dataset, corresponding to a scan of a methylene blue dyed esophagus. (**a**) Schematic of the process of acquiring a video from the pig esophagus. Translation of the endoscope is done by free-hand movement. (**b**) Registered frames from a left-to-right scan of the pig esophagus, corrected for the fiber artifacts and registration artifacts. (**c**) Output of the classification, for the slices (i,ii) highlighted on the prediction map and the registered image. (**d**) Prediction map of the scanned area.

As a first step to understand how the color classification approach might translate into a tissue application, the HySE system was deployed in an intact *ex vivo* pig esophagus (Fig. 4a). The resulting video was classified on a frame-by-frame basis, with an achievable speed of ~7 fps. Comparing the reconstructed wide-field reference image (Fig. 4b) with the CNN classification result (Fig. 4c) shows the structure of the lumen (black hollow), unstained tissue (pink) and methylene blue dyed tissue (blue). Slice-based classification overlaid on the wide-field imaging data shows similar results (Fig. 4d). Unlike in the validation experiments, dark streaks can be seen in the classification result, despite the fact that there is no typical background in the pig esophagus dataset. The background class is identified in the region of the lumen, where the long working distance means that the signal is too low for the color to be correctly classified. Dark streaks due to identification of the background class also appear at interface regions between two different colors (i.e. pink and blue). A potential reason for this phenomena is that the mixture of different colors lowers the certainty of the algorithm towards a single color class, leading to it being classified instead as background.

### Tissue biospy dataset

**Figure 5:**
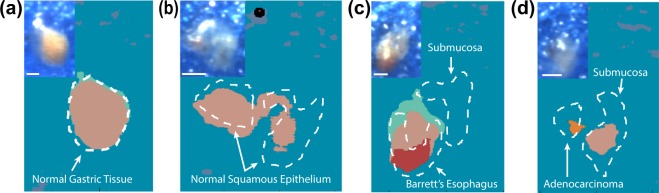
CNN color classification results for human esophageal tissue biopsy samples. White-light RGB images of each tissue biopsy reconstructed from the hypercube are shown in the top-left corner of each classification map. Dashed lines show the endoscopist annotation based on histopathological analysis for the different biopsy samples (**a**) Normal gastric tissue. (**b**) Normal squamous epithelium. (**c**) Barrett’s esophagus. (**d**) Adenocarcinoma. Scale bars = 1 *mm*.

To test the capability of the color classification approach to differentiate between subtle spectral changes, we investigated the performance on a series of esophageal tissue biopsies exhibiting tissue types ranging from normal mucosa to cancer. For this experiment, four different tissue classes were employed: gastric epithelium, normal squamous epithelium, Barrett’s esophagus and adenocarcinoma. Tissue classes were determined by standard-of-care histopathology. Examples of a biopsy tissue from each class and their respective classification maps are shown in Fig. 5. For comparison with the CNN predictions, the decision boundaries drawn by the operating endoscopist are also shown (see Methods). As would be expected based on the generally pink color of the esophagus, the color classifications (again obtained at ~7 fps) are identified as those closest to pink within the color chart. Encouragingly, the classification colors for regions of normal gastric (Fig. 5a) and squamous (Fig. 5b) epithelium, Barrett’s esophagus (Fig. 5c) and adenocarcinoma (Fig. 5d) are distinct, likely due to the differences in vascularity and hence hemoglobin concentration in these regions^4^. We obtained a 86.9 % average consistency of classification in the tissue types with multiple samples (Barrett’s esophagus, gastric and squamous epithelium), with consistency being calculated as the union of the color area with the decision boundary, with background coloured regions excluded. The physical areas that were successfully classified as tissue appear to be underestimated by the CNN compared to the endoscopist, however, this is most likely due to the transparency of the sample edges, which has led the CNN to classify according to the color of the background material upon which the biopsies were laid. Nonetheless, the distinct classifications of the different tissue in this preliminary analysis  suggests promise of the color-based CNN to enable interpretation of HySE data by the operator during real-time operation.

## Discussion

The application of HSI in real-time biomedical imaging, for example during endoscopy, requires rapid processing of a high volume of hyperspectral data in order to provide relevant feedback to the operator. Here, we introduce a five layer CNN based on color classification as a means to facilitate such feedback. Our results show that pixel-wise classification of hyperspectral endoscopy data based on 18 pure color spectra is possible with high discrimination accuracy. The color-based classification not only performs well in the planar imaging case, but also maintains good performance when applied under conditions of variable working distance. We tested the approach in "unseen” data acquired under somewhat more realistic conditions, including data from an intact pig esophagus and human esophageal tissue biopsies *ex vivo*. Color-based classification was able to distinguish areas of the pig esophagus containing methylene blue dye from the undyed background tissue, although misclassification of tissue areas as background arose at the boundaries between the regions. Encouragingly, however, when applied to preliminary data from human tissue biopsies representing normal gastric and squamous epithelium, Barrett’s esophagus and esophageal adenocarcinoma, the classification was able to separate these tissue types into different colors.

While the results presented show promise for the real-time application of color-based classification, two key limitations that need to be overcome towards practical implementation. Firstly, while the accuracy of the system is high, even in the planar dataset the maximum color classification accuracy was 94.3%. The loss of accuracy arises primarily due to misclassification at the edges of the color squares. This may be due to the combined effect of: the spatial averaging taking place within the line-scanning slit of the HySE system; and the presence of cladding artifacts from the multi-core fibre bundle. Some pixels at the edges of the color squares thus contain a mixed contribution of the color class and the background. A more prominent example of this effect is seen in the pig esophagus data, where black streaks are present throughout the classification map that are attributed to the same effect. In this case mixtures of multiple colors may be present within the misclassified regions so one color does not gain enough weight to pass the classification threshold, leading to it being viewed as an unlabeled class, different from the ones for which the network was trained. This was again seen in the tissue biopsy data, where the physical areas of the image that were successfully classified as tissue appear to be underestimated by the CNN compared to the indications of the endoscopist, most likely due to the transparency of the sample edges. To solve this problem in future, we could expand the number of colors on which the CNN is trained, including color charts with a greater proportion of red and brown colors. We could also further develop the algorithm from a pure classification of colors to the generation of abundance maps for the contribution of each of the 18 colors. To do so requires a change in the final layer of the network, such that abundances are kept proportional to the weights of the colors. Nonetheless, changing to a probability-based map would require additional steps for classification, which would likely lead to a decrease in the overall classification speed.

The second limitation of the color-based classification is the current classification speed (around 7 fps), which is lower than the hyperspectral data collection speed of the HySE system (20 fps)^4^. In the initial implementation of the pixel-wise CNN, the limiting factor in data classification is the preprocessing time, which constitutes 80% of the overall algorithm run-time. By further streamlining the preprocessing stage, the frame rate could be double, but would still be lower than the imaging speeds achieved by the HySE system. Nonetheless, these values are similar to classification rates achieved in other studies dealing with the online classification of hyperspectral data (e.g. 12 fps^14^). To address the discrepancy between the classification and imaging speeds, the refresh rate of the color classification display could be reduced compared to the wide-field color reference image. Future implementations towards real-time operation could aim to further improve the classification speed by circumventing collection of the data from the computer memory by receiving and processing data directly from the camera itself. A further limitation of the study is that the evaluation of the pig esophagus and human esophageal biopsy data sets was purely qualitative due to the absence of an appropriate gold standard. In the case of the pig esophagus, the dye application could not be confined to a particular area, so without opening the esophageal lumen, it was not possible to identify solely from the wide-field color image precisely where the blue dye was located. For the tissue biopsy specimens, the histopathological analysis gives only a single decision for the highest grade of pathology found within the whole specimen. Therefore a spatially resolved classification, such as that performed here, cannot be linked to the precise pathology contained within each pixel.

To truly establish the performance of the color-based classification in a clinically realistic scenario, hyperspectral data should in future be acquired from samples in which a more fine grained histopathological ground truth is available, for example, in mapped endoscopic mucosal resections^18,19^. Once available, the CNN could be more thoroughly tested and also further tailored for application in the discrimination of early cancer. For example, finding implementations that can predict spectral mixture proportions rather than pure spectra (as mentioned above) could then be used for generating characteristic spectra of the various stages of adenocarcinoma in the esophagus. Those characteristic spectra would not only serve as guidance during HySE operation, but could also be useful in future for determining the main biochemical components that can lead to such differences in early disease.

## Methods

### Optical setup and data acquisition

The line-scanning hyperspectral endoscopy (HySE) system used for data acquisition in this study has been reported previously^4^. Briefly, HSI data was acquired via a line-scanning spectrograph (IsoPlane 160, Princeton Instruments) coupled with a CCD (ProEM CCD, Princeton Instruments), which measures spectral (*λ*) information along a single spatial line (*y*) (Fig. 1a,b). The spatial information from the second axis (*x*) is composed during motion of the endoscope, which is either achieved with freehand movement (as per normal endoscopic operation) or for our phantom studies, using a motorized translational stage (MTS50/M-Z8, Thorlabs). A 300 lines/*mm* or a 50 lines/*mm* grating, with spectral bandwidths of 125 *nm* and 750 *nm* respectively, were employed in this study. In order to measure a spectral image across a wide range of wavelengths for the 300 lines/*mm* grating, data acquired at center wavelengths of 450, 550 and 650 *nm* were merged into a single spectral image. Wide-field color images were acquired using a CMOS camera (GS3-U3-51S5C, Point Grey Research). Wide-field color images can either be viewed directly with the classification resulting from the spectral information overlaid in the measured line, or used to prepare a panoramic image by geometric transformation and image co-registration (Fig. 1a). For initial testing, the geometric transformation matrices were applied to reconstruct a fully co-registered 3D hypercube. Gold standard reference spectra were acquired using a second spectrometer (AvaSpec ULS2048L, Avantes). Samples were illuminated using a halogen light source (OSL2, Thorlabs) with a light bulb (OSL2bIR, Thorlabs) whose emission spectrum spanned across visible to NIR (400 to 750 *nm*). The fiber-coupled light source was positioned 10 *cm* away from the sample, thus providing a static wide-area illumination at high power across the whole imaging area.

### Training data

**Figure 6:**
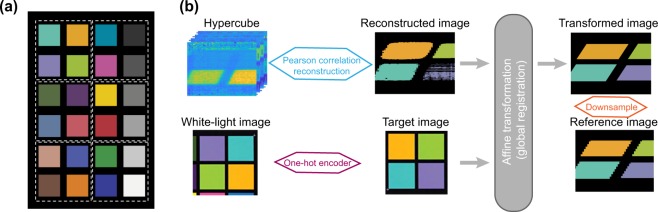
Generation of training and reference data. (**a**) Illustration of the Macbeth color chart. Six separate 25 *mm* ×  25 *mm* sections of the color chart, each consisting of four target colors, were imaged in duplicate to obtain training hypercubes (**b**) Schematic for the generation of the reference images.

Training data for the study was provided by imaging a Macbeth color chart (ColorChecker Classic Mini, x-rite), a color calibration target consisting of 24 squares of painted samples, 18 of which contain colors whose spectral reflectance is intended to mimic those found in natural objects and 6 of which are a uniform gray lightness scale. The training data was constructed using 2 independent experimental measurements of the full color chart. During each experimental measurement, the color chart was divided into 6 separate 25 *mm* ×  25 *mm* sections, each consisting of four target colors, with the dimension of a single hypercube (*x*, *y*, *λ*) recorded from a given section being (50, 512, 1209) (Fig. 6a). A total of 300 hypercubes were then generated by augmenting these initial 12 measured hypercubes through the addition of further Gaussian noise and linear illumination variations, with biases being collected from a distribution of mean 0.1 and the randomly inclined slopes being collected from a distribution of mean 0.01. Standard deviations for the two distributions are 0.04 and 0.03, respectively. We introduced a slight bias towards under-illuminated scenarios as these are commonly encountered in endoscopy. A five-fold cross validation process was employed when selecting the training and validation data from the 312 hypercubes. To compare the performance of the deep-learning based approach to more traditional multivariate statistical methods, algorithms were first trained on a 4 color subset of the training data, consisting of data from one section of the color chart. Following this, the convolutional neural network was trained to recognize the 18 colors of the chart, with the 6 grayscale targets being excluded.

To evaluate the classification accuracy of different spectral analysis methods, reference spectra from each of the color chart squares, and reference images that reflect the spatial data acquired by HySE from the color chart, were then needed. For the reference spectra, an optical fiber coupled to the reference spectrometer was placed directly above the color chart square, leading to the acquisition of 18 color spectra with a spectral resolution of 0.6 nm and 513 spectral pixels. The spectrometer was run with a 14.32 ms integration time and averaged over 35 scans, leading to a measurement time of 0.5 s. During the HySE imaging process, co-registered wide-field color images and line-scan spectral images are acquired, so the wide-field color images were used as the image reference. A landmark-based affine geometric transformation was employed to account for differences in the resolutions and orientations the two cameras and the process was optimized based on a Dice similarity coefficient^20^. Finally, due to the co-registered image being of higher resolution than the classification output, it was down-sampled for direct comparison with the classification results, leading to the generation of the reference image (Fig. 6b).

### Data preprocessing

After the acquisition of the hypercube from a given sample, a standard reflectance target (LabSphere) was measured under the same experimental conditions to provide a white reference hypercube for normalization purposes. To obtain exact reflectance signals and to assure algorithmic efficiency, the hyperspectral data was normalized to subunitary values, using this reference target data, *I*_*white*_, according to:

1 $${I}_{n}=\frac{I-{I}_{dark}}{{I}_{white}-{I}_{dark}}$$

where *I*_*n*_ is the normalized reflectance, *I* is the measured intensity and *I*_*dark*_ is the dark signal measured from the sensor. Furthermore, to remove "salt and pepper” noise, which can skew the classification results, a 7  ×  7 2-D median filer is applied across the hypercube.

### Data classification methods

We selected 3 conventional spectral analysis methods to compare to the learning-based method established here. Due to the data of the different datasets having variations in both the spatial sizes and spectral sizes, we chose methods that would be robust to variations in dataset dimensions to maximize flexibility during testing.

#### Pearson correlation analysis

Calculates an index of linear dependence of the HySE spectra and the reference spectra recorded by the second spectrometer. For two spectra *A* and *B*, the Pearson correlation coefficient (*ρ*) is defined as:

2 $$\rho (A,B)=\frac{1}{N-1}{\sum }_{i=1}^{N}\left(\bar{\frac{{A}_{i}-{\mu }_{A}}{{\sigma }_{A}}}\right)\frac{{B}_{i}-{\mu }_{B}}{{\sigma }_{B}}$$

where *N* is the number of samples, *μ*_*A*_ and *σ*_*A*_ are the mean and standard deviation of *A* respectively, and *μ*_*B*_ and *σ*_*B*_ are the mean and standard deviation of *B*^21^.

#### Spectral unmixing

Assumes the spectrum in a given pixel is a linear combination of the reference spectra recorded by the second spectrometer. Spectral unmixing determines the relative contribution of the different reference spectra to the recorded signal. Both supervised and unsupervised unmixing algorithms were tested, with the supervised technique consisting of a linear regression algorithm and the unsupervised technique being based on the non-negative matrix factorization technique developed by Bioucas-Dias *et al*.^22,23^.

#### Support vector machines

Are binary classifiers that optimize hyperplanes between two data populations of interest, with the best hyperplane being the one with the highest achievable margin. A one-versus-one classification approach was chosen for the multi-class problem under study here^24^.

#### Convolutional neural networks

Were trained in both a pixel-wise and slice-wise manner. The CNNs were implemented in Python, with Theano libraries being used to access graphical processing unit (GPU) acceleration. To provide robustness to variations in the input spectral resolution (arising, for example, from the use of different gratings), a 121 equispaced samples binning function was applied to the input hypercube before testing. For the pixel-wise CNN, the spectrum of each spatial pixel was wrapped into an 11  ×  11 pixel spectral patch, which was then fed into the network together with the categorical labels, one-hot encoding the color chart colors, accessed from the reference images. The wrapping technique introduces prior knowledge on the correlations of interest in the spectrum, thus allowing the use of a network with two convolutional layer. This allows the training of a classifier with less data, as high quality labeled data is difficult to acquire in the optical imaging of biological tissue.

**Figure 7:**
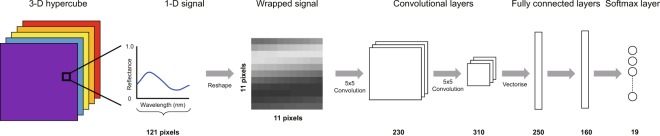
Diagram of the CNN architecture. Number and sizes of the convolutional filters are annotated on the diagram.

The network architecture consists of two convolutional layers and two fully connected layers, with rectified linear unit (ReLU) activation functions^25^ being employed throughout the network (Fig. 7). The CNN was trained using a mini-batch stochastic gradient descent (SGD) back-propagation algorithm with a momentum-based learning rate. Training was done on mini batches of 100 data points at a momentum adjusted learning rate of 0.001 for 900 epochs or until the early stopping condition. Internal accuracy for the SGD was determined using:

3 $${\rm{argmax}}\,E\ne {\rm{argmax}}R$$

where *E* represents the classified endoscopy results and *R* represents the reference label. In addition to the ReLU activation function, a 40% dropout rate and L2-regularization are employed to prevent overfitting. A softmax activation function is implemented as a decision layer, with a nineteenth background class being employed as a label for any spectra which do not match those found in the color chart. Testing of the system was done on a GPU machine (GeForce GTX 1060, 16 GB RAM).

A slice-wise CNN architecture was also tested as an alternative to the pixel-wise classification algorithm due to the fact that the HySE system acquires line-scan hyperspectral data. This alternative implementation takes the raw 2-D spatial-spectral images as the input. The slice-wise CNN architecture consists of four convolutional layers and two fully connected layers, with max-pooling and ReLU activation functions being implemented.

### Performance evaluation

Performance of the analysis methods was measured based on the per-slice classification time and the average classification accuracy throughout the fully recorded hypercube. The classification accuracy (ACC_*av*_) is calculated following:

4 $${{\rm{ACC}}}_{av}=\left(1-\frac{\sum ma{x}_{L}| {E}_{L}-{R}_{L}| }{N}\right)\times 100$$

where *max*_*L*_ represents the maximum value along the class dimension of the matrix, *N* is the total number of pixels, *E*_*L*_ represents the classified endoscopy result in categorical format and *R*_*L*_ represents the reference image in categorical format.

### Pig esophagus preparation

A fresh *ex vivo* pig esophagus and stomach (Medical Meat Supplies) was used as described previously^4^ to mimic the imaging conditions during endoscopy, including video rate data acquisition and data acquisition in a narrow lumen. Hyperspectral endoscopy was performed using a grating of 50 *lines*∕*mm* and exposure time of 25 *ms*, with motion from right to left sides of the lumen used to build up the second spatial dimension of information. Methylene blue dye (319112, Sigma-Aldrich) was sprayed inside of the lumen to induce a color change for identification with our color-based classification approach.

### Tissue biopsy preparation

Tissue biopsy samples were collected at Addenbrooke’s Hospital from patients (number of patients = 3; number of biopsies = 12) undergoing diagnostic work-up or endoscopic therapy for Barrett’s-related intramucosal esophageal adenocarcinoma. Ethical approval for the study was received by the Cambridgeshire 2 Research Ethics Committee (09/H0308/118). All research was performed in accordance with relevant guidelines and regulations, with informed consent being obtained from all patients. Endoscopic mucosal resections were performed on suspicious areas and these then sampled *ex vivo* using a 2 *mm* diameter biopsy punch. Collected samples were positioned in individual containers, with the epithelial layer facing upward. Soft sheets of blue sponge were added to the containers to minimize sample movement during transportation. Autoclaved phosphate-buffered saline was added to the sample and sponge, to keep them hydrated during the HSI procedure. Tissue biopsy datasets were collected over an area of 5 *mm* ×  5 *mm* (motorized stage step size of 50 *μm*) over a time period of 150 *s* (exposure time per step of 500 *ms*). Sample measurements were completed within 3 *h* of the biopsy being taken to minimize biological variation due to removal from the patient. All biopsy samples were then subjected to histopathological analysis and to obtain the gold standard of diagnosis. Visual interpretation by the endoscopist, informed by histopathology, was provided to generate the tissue outlines on our HSI data (white dashed lines in Fig. 5).

## Data Availability

All data will be made openly available upon publication of this manuscript. 10.17863/CAM.49542.
